# Rapid Convolutional Neural Networks for Gram-Stained Image Classification at Inference Time on Mobile Devices: Empirical Study from Transfer Learning to Optimization

**DOI:** 10.3390/biomedicines10112808

**Published:** 2022-11-04

**Authors:** Hee E. Kim, Mate E. Maros, Fabian Siegel, Thomas Ganslandt

**Affiliations:** 1 Department of Biomedical Informatics, Center for Preventive Medicine and Digital Health (CPD-BW), Medical Faculty Mannheim, Heidelberg University, Theodor-Kutzer-Ufer 1-3, 68167 Mannheim, Germany; 2 Chair of Medical Informatics, Friedrich-Alexander-Universtät Erlangen-Nürmberg, 91054 Erlangen, Germany

**Keywords:** Gram-stained classification, deep learning, model compression, pruning, quantization, rapid inference time, mHealth

## Abstract

Despite the emergence of mobile health and the success of deep learning (DL), deploying production-ready DL models to resource-limited devices remains challenging. Especially, during inference time, the speed of DL models becomes relevant. We aimed to accelerate inference time for Gram-stained analysis, which is a tedious and manual task involving microorganism detection on whole slide images. Three DL models were optimized in three steps: transfer learning, pruning and quantization and then evaluated on two Android smartphones. Most convolutional layers (≥80%) had to be retrained for adaptation to the Gram-stained classification task. The combination of pruning and quantization demonstrated its utility to reduce the model size and inference time without compromising model quality. Pruning mainly contributed to model size reduction by 15×, while quantization reduced inference time by 3× and decreased model size by 4×. The combination of two reduced the baseline model by an overall factor of 46×. Optimized models were smaller than 6 MB and were able to process one image in <0.6 s on a Galaxy S10. Our findings demonstrate that methods for model compression are highly relevant for the successful deployment of DL solutions to resource-limited devices.

## 1. Introduction

The number of mobile health (mHealth) apps is growing substantially. The number of mHealth apps in the Google Play store reached over 54,603 in the second quarter of 2022 [1], while there were 52,406 in the Apple App Store [2]. According to Roth [3], mHealth apps can be classified into four categories: information apps, which provide a recent trend in healthcare and allow users to find medical information; diagnostic apps, which process data to support physicians in diagnostic decisions; control apps, which control basic functionalities such as the power switch of another medical device; and adapter apps, which adapt smartphones to perform a medical function.

The application developed and evaluated in this study is a diagnostic app, which automates Gram-stained analysis. It is a laboratory procedure that classifies microbial pathogens as either Gram-positive or Gram-negative. It is a promising application in a microbiology laboratory because this task still relies on humans. Physicians and trained medical technical assistants need to navigate the whole slide images manually. This problem can be leveraged by recent advances in deep learning (DL) methodologies, in particular, convolutional neural networks (CNN) which have emerged as the de facto DL methodology in the field of image analysis. For instance, a whole slide image can be distinguished into major species of microorganisms or the position of microorganisms can be highlighted directly on the image. In this manner, the system could enhance the competencies of caregivers with less human intervention. This could lead to a rapid initial medical care for patients who suffer from infectious diseases.

However, deploying a DL solution is a non-trivial problem and deploying to resource-limited and battery-powered devices such as smartphones is challenging. For instance, Smith et al. reported that it took 9 min to classify a single whole slide image with a workstation powered by Nvidia GTX 1070 GPU [4]. Moreover, Netflix announced in 2012 that they failed to deploy the winner solution of the “1 million-dollar Netflix Challenge” due to engineering costs of the complex machine learning solution [5]. One of the major obstacles is the computational burden because DL models consist of millions of parameters. For instance, ResNet152 and AlexNet models consist of 60 million parameters and 132 million parameters for the VGG16 model, meanwhile, the latest Google glass enterprise Edition 2 released in May 2019 features only 3 GB of memory, 32 GB of storage and 800 mAh battery capacity, allowing for only 8 h of running time. Accordingly, resource utilization becomes a non-trivial issue because millions of arithmetic operations require longer processing time and drain the battery more quickly. Especially, battery-powered devices (e.g., mobile devices, internet of things and wearable devices) must be carefully considered when DL solutions are developed.

This challenge has led to compact and rapid DL as an emerging topic in recent years. Han [6] distinguished four types of research endeavors on this subject based on what and how to speed up DL models. The target to be accelerated is either training time or inference time; on the other hand, it can be achieved by introducing novel hardware or tuning algorithms optimally. Graphics processing unit (GPU) initially developed for accelerating computer graphics is now a core element of server infrastructure for the rapid deep learning processing. Google developed an application-specific integrated circuit (ASIC) known as a tensor processing unit (TPU) [7], which is optimally designed to process deep learning solutions implemented by its own framework, TensorFlow. Within the realm of efficient algorithms, numerous approaches have been proposed; for example, Chollet et al. [8] speeded up the training time with little accuracy degradation by introducing an innovative model architecture with depthwise separable convolutional neural networks. Smith et al. [9] and Goyal et al. [10] shortened training time by applying a large batch size (BS). Numerous normalization approaches [11,12,13,14] and regularization techniques such as early stopping [15] and structure sparsity regularization by suppressing irregular memory access successfully accelerated training time. On the other hand, model compression methods such as pruning [16] and quantization [17] are able to expedite the inference time. Pruning removes the low-impact parameters incrementally, while quantization scales down the bit representation from 32-bit floating-point numbers to lower-bit representation.

The contributions of this study are as follows: identify the optimal transfer learning configuration of CNN models to Gram-stained image classification; accelerate the inference time by model optimization methods; and deploy and evaluate the execution speed of the optimized models on two Android devices.

## 2. Materials and Methods

### 2.1. Efficient Convolutional Neural Networks

CNN [18] is a class of deep neural networks that are designed to solve various computer vision problems. CNN constitutes fully connected layers and convolutional layers. The former is the classical layer where all neurons are interconnected to one another in adjacent layers, while the latter is a core element of CNN which generates generic feature maps from the previous layer. In terms of computational complexity, the convolutional layer is less expensive because neuron weight sharing reduces the number of connections between neighbor layers.

**Figure 1:**
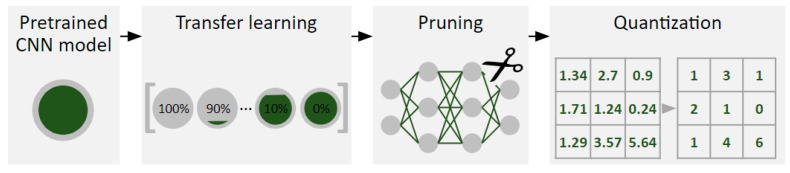
A flowchart diagram depicts the process to optimize naïve CNN models to the efficient CNN models. Transfer learning adapts CNN models pretrained from natural images to the custom image dataset. Pruning trims out non-significant weights while quantization drops floating-point numbers by rounding a given value to the nearest integer number.

The pretrained CNN models are tuned to the efficient models in three steps. The overview is illustrated in Figure 1. The first step is transfer learning (TL), which is a technique particularly widely adopted technique for medical image analysis owing to its capability of model adaptation towards new tasks [19]. TL is inspired by the learning mechanism, in which the knowledge acquired before can leverage the learning procedure to learn similar tasks. Since TL can reuse weights of pretrained CNN models, TL is able to reduce the computational burdens to a large degree. Pruning zeros out non-significant connections in neural networks. It gradually eliminates low-impact parameters based on magnitude without decreasing model accuracy. Unlike dropout [20], it ignores some nodes randomly during the training phase but pruning eliminates model parameters (connections). This attribute makes models require less storage overhead and reduces the memory footprint. Quantization converts 32-bit floating-point numbers to lower-bit representations such as 8-bit integer numbers. An intuitive example of quantization is converting floating-point numbers to integer numbers (e.g., 1.245 to 1). Unlike pruning being applied during the training phase, quantization is a post-production method because it is typically applied during the post-modeling phase.

### 2.2. Data Set

**Figure 2:**
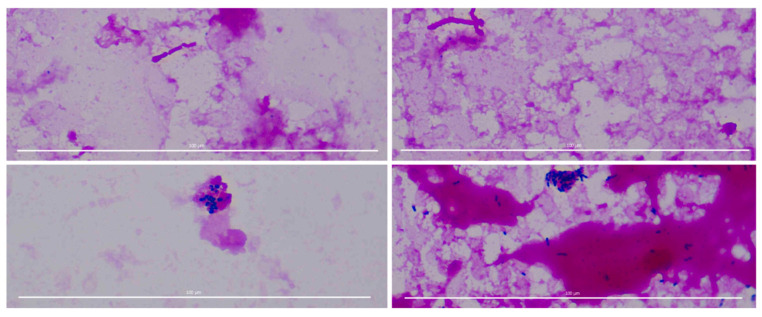
Sample images of Gram-stained data. Two Gram-negative images are shown on the top, and two Gram-positive images are shown on the bottom. Some pathogens are distinctive with a high contrast of a clean background whereas often other pathogens are blurred and/or have bloodstains in the background and/or low brightness level. Scale bar represents 100 µm.

Eight thousand five hundred Gram-stained images with two labels (positive vs. negative) were taken from sepsis patients who suffered from at least one microbial infection such as Staphylococcus, Escherichia, or Streptococcus. Images with both labels (two types of germs appeared on a single image) were excluded from this study (*n* = 446) in order to make a binary image classification. Given images were cropped areas of interest containing stained microorganisms from a whole slide microscopy image. The size of the images varied from 800-pixel by 600-pixel to 1920-pixel by 1080-pixel. Exemplary sample images and labels are shown in Figure 2.

Gram-positive images were two-fold more frequent (*n* = 5962) than Gram-negative images (*n* = 2766). Therefore, class balancing needed to be applied. Otherwise, the models were conditioned to predict the majority labels and abandon the minority class. Hence, Gram-negative images were augmented to balance the class proportion by rotating the given images. After the augmentation, the dataset was enriched from 8728 to 10,994 images. For the sake of a fair evaluation, the test dataset and validation dataset was isolated from the training set. This study split the given data into 80% for training, 10% for validation and 10% for testing.

### 2.3. Study Design

The machine learning task in this study is binary image classification. The implemented models will predict whether the image is Gram-positive or -negative. Three pretrained models were utilized in order to avoid model selection bias. They are, namely, Inception [21], ResNet [22], and MobileNet [23]. Inception was chosen because it is the most prevalent model utilized in the medical domain according to Morid et al. [24] and Kim et al. [19]. Furthermore, ResNet is the most widely used backbone model for other tasks such as object detection and segmentation [25]. Finally, MobileNet was selected because it was explicitly designed to be deployed to resource-constrained-devices [23]. Each model was calibrated to the Gram-stained analysis and then optimized and evaluated. The consecutive steps performed are as follows: TL; pruning; quantization; and evaluation.

The main objective of TL is to identify the best accuracy setup and others are to reduce the model size and minimize the inference time without accuracy loss. Primarily, pretrained models were tuned to Gram-stained images because the given models were trained with the ImageNet dataset [26] containing natural images only. The optimal fine-tuning ratio was determined by exploring numerous configurations. The number of model layers was binned into 10 buckets and each bucket was incrementally fine-tuned from the shallow strategy (feature extraction) to the deep strategy (fine-tuning from scratch). The former strategy is also referred to as feature extraction and it updates no convolutional layers except the fully connected layers, while the latter updates all layers from scratch. This study iteratively walked through 11 different settings from the shallow strategy (retraining 0%) to the deep strategy (retraining 100%).

Once models were transferred to Gram-stained images, one of the model compression methods, pruning was applied. Pruning trims the low impact parameters incrementally. In other words, model parameters were iteratively pruned from 10% up to 90%. Similar to the fine-tuning method, nine target sparsity values were evaluated gradually from the dense model (pruned 10%) to the very sparse model (pruned 90%). Following this, another model compression method, quantization was applied. In this study, we scaled down the default 32-bit representation to three lower bit-schemes, namely 16-bit floating-point numbers, 16-bit mixed numbers (floating and integer) and 8-bit full integer numbers.

### 2.4. Metrics

Accuracy evaluates the quality of models; however, it fails to provide insight into model behaviors when it is deployed to production. Computational costs and model size should be considered especially when it is deployed to resource-constrained devices. Hence, this study evaluated models not only with the classical metrics (accuracy) but also with model size and inference time. For the sake of statistical stability, model accuracy was tested 10 times while inference time was tested 50 times and the average values were reported in this paper.

### 2.5. Apparatus

Tensorflow and Tensorflow Lite were the chosen frameworks for deep learning solutions in this study. Both frameworks are open-source tools developed by the Google Brain team [27]. TensorBoard was used as a model-debugging tool and to graphically track all execution history.

All models were processed at the data center of the Department of Biomedical Informatics at the Center for Preventive Medicine and Digital Health Baden-Württember, Medical Faculty Mannheim. Regarding the reproducible research, hardware was virtualized by Docker for a controlled development environment. Each container was configured with one Intel Xeon Silver 4110 CPU, one NVIDIA Tesla V100 32 GB GPU and 189 GB of shared memory.

The inference time of the compressed models was evaluated on two android mobile devices: Samsung Galaxy A20E and S10. The quantized models need to be tested on devices with ARM-based CPU and not x86-based CPU workstations because integer arithmetic is optimized for the ARM CPU architecture. The averaged inference time was measured by a C++ binary tool developed by Google via a command-line interface called Android Debug Bridge allowing communication with mobile devices. This study utilized only one CPU thread on mobile devices. All other active processes were deactivated during the testing, and the network state was switched off.

## 3. Results

### 3.1. Transfer Learning

**Figure 3:**
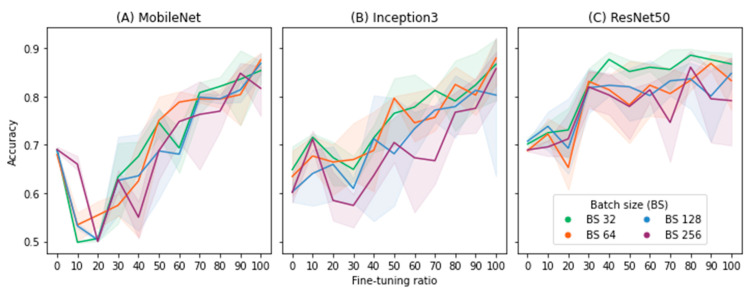
Result of test accuracy based on the combination of 11 tuning ratios and four batch sizes (BS). Each setup/ratio has been repeated and tested 10 times for the sake of statistical analysis. The average is shown as a bold line, while the minimum and maximum accuracy are shown as areas in a lighter color.

Twelve models (three models with four different batch sizes) were evaluated. Figure 3 illustrates the results of the three pretrained models. Regardless of model and batch size, there was a noticeable trend shown in Figure 3 in which accuracy dropped when only a few layers were retrained (approximately 10 to 20% of the total number of layers of the respective model/architecture), but it recovered when more layers (>50%) were retrained. The highest accuracy for Inception3 and MobileNet was achieved when the model was retrained from scratch (100%) with 64 minibatch, while ResNet50 attained the best accuracy with the fine-tuning ratio of 80% and 32 minibatch.

All execution histories were reported in our GibHub repository and they are publicly accessible at: https://github.com/kimheekimi/rapid_gram_stain/tree/main/results (accessed on 1 November 2022). The average training time for TL was roughly 145 min (220 min for ResNet50, 160 min for Inception 3, and 60 min for MobileNet) when the number of epochs was 100. The exact training time was not reported in this section because the scope of this paper was to compare the inference time.

### 3.2. Pruning

**Figure 4:**
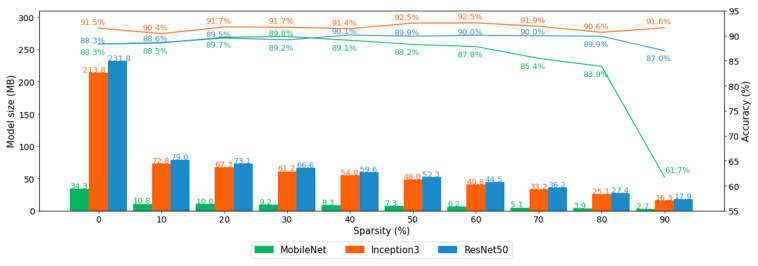
The results of models with 0% sparsity (leftmost) are the baseline where pruning was not applied. The bar chart depicts that all pruned models except MobileNet were successfully compressed without sacrificing the model accuracy shown in the line chart. The accuracy deteriorated when MobileNet pruned more than 70%.

Twenty-seven models (three models with nine different pruning ratios) were pruned and evaluated in this phase. Each setup was trained and tested 10 times and the averaged accuracy values are depicted in Figure 4. The result shows that pruning was able to compress the model up to 15 times (Figure 4. Bar chart) as compared to the baseline model (0% sparsity) without or with only a minor loss of model accuracy (Figure 4, line chart). Only MobileNet (colored in green) with a high sparsity ratio suffered from a substantial decrease in accuracy.

### 3.3. Quantization

**Figure 5:**
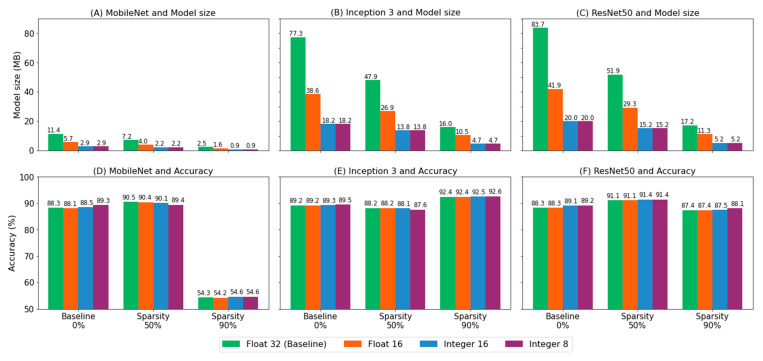
Quantization method reduced model size (**A**–**C**) with minor accuracy loss (**D**–**F**). Models in 16-bit float type were 2 times smaller than the baseline model and models in integer-type were 4 times smaller than the baseline model.

The weights and activations of pruned models were converted from 32-bit float to 16-bit float, 16-bit integer and 8-bit integer numbers. Accuracy was not dropped for all models despite the model size having been significantly reduced. Figure 5 shows that the size of models converted to integer-type was reduced from at least 3 times and up to 4.3 times (Figure 5A–C) with accuracy loss at most 1.1% to accuracy gain up to 0.9% (Figure 5D–F).

### 3.4. Evaluate Inference Time on Mobile Devices

**Figure 6:**
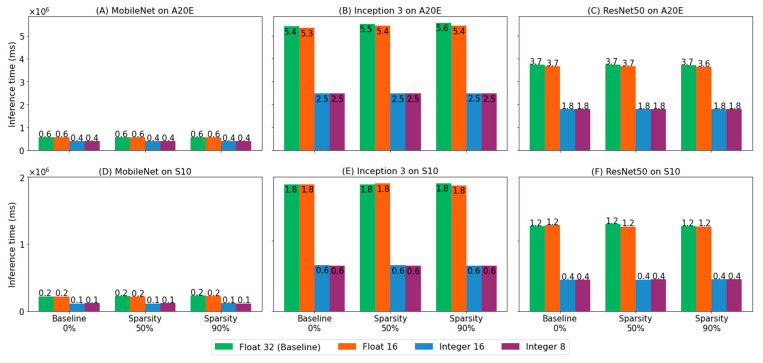
Inference time on Galaxy A20E and S10. The latency of integer-type models (blue and purple) is at least 1.9 times faster and at most 2.8 times faster than the float-type models (green and orange).

The three clusters represent different pruning ratios of models from 0% to 50% to 90%, as shown in Figure 6 (*x*-axis). The leftmost cluster is the baseline model to which pruning was not applied. On a cluster-to-cluster basis comparison, there was no remarkable difference among clusters in terms of the inference time. The latency of 50% and 90% sparse models on mobile devices were similar to that of the baseline model.

Each cluster consists of four bars in four colors indicating different bit schema from float 32, float 16 and integer 16 to integer 8. On a bar-to-bar basis comparison, quantization sped up the inference time to at least 1.9 times to 2.8 times faster. The improvement of the execution time was more distinctive on Galaxy S10 than A20E.

## 4. Discussion

The performance of the fine-tuning method was not much influenced by batch size. An empirical study by Wilson et al. [28] stated that a large batch size leads to a decrease in performance; however, we did not observe a significant accuracy drop in this study. In fact, a large batch size requires fewer iterations to converge the respective model at the expense of using more memory, but it was only a marginal gain (2 to 5 s faster) in training time. On the other hand, the performance was highly sensitive to the fine-tuning ratio due to the heterogeneity of features between Gram-stained images and natural images. In this study, the highest accuracy was attained by retraining convolutional layers by at least 80%. Hence, in order to capture the characteristics of Gram-stained images, we recommend retraining as many model layers as possible.

**Figure 7:**
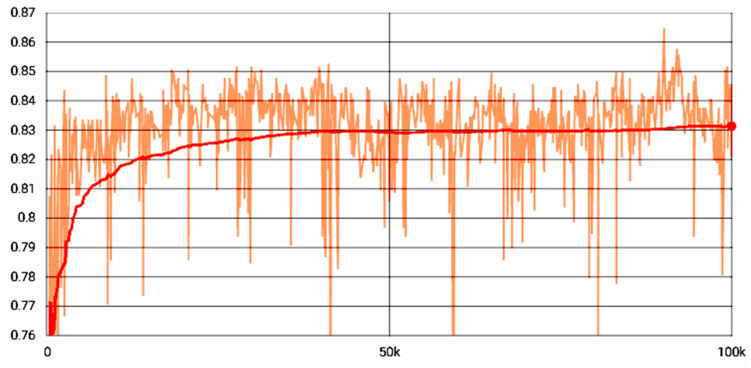
MobileNet with 90% sparsity was trained for 100,000 epochs. Accuracy recovered (83%), but it was not as good as the baseline model (88%). The noisy data points were smoothed by a moving average method, which calculates a series of averages of subsets of data points.

Model size and accuracy were affected by the pruning method to a large margin as shown in Figure 4. Although the model comprised many fewer parameters, pruning did not decrease the model accuracy, except for the MobileNet. The ResNet50 model with 90% fewer parameters was 13 times smaller than the baseline model; nonetheless, the accuracy increased by a small margin. However, the 90% sparse MobileNet suffered from low accuracy as it dropped to 61.7% (Figure 4, Line chart). We further investigated with the 90% pruned MobileNet whether the accuracy can be recovered by extending the training steps. For this, we trained the model for 100,000 epochs which took 36 days at the workstation described in the Method-Apparatus section. Figure 7 shows that the accuracy recovered from 76% to 83%; however, it would be hard to justify such extensive training for only marginally better accuracy.

Similar to pruning, quantization reduced the model size up to 4.3 times without losing accuracy. To our surprise, both quantized and pruned models occasionally gained accuracy by a small margin. We assume that removing unnecessary parameters and lowering bit-representation might restrict the DL models not to overfit the training dataset. With regard to the inference time, no significant differences were reported among the same data type (e.g., float 32 and float 16; integer 16 and integer 8). It is because the major operations (matrix multiplication and backpropagation) are still carried out using 32-bit in spite of the input and output being quantized into lower bit representation. Matrix multiplications process multiple 8-bit or 16-bit operands that require more bits to process and store. On the other hand, backpropagation with a lower bit could not nudge the subtle updates for weights and biases. Both accumulator and backpropagation are the cornerstone tasks of convolution and therefore require more computational costs.

The inference time on the smartphone Galaxy S10 was more distinctive than the smartphone Galaxy A20E, as illustrated in Figure 6. The major reason is cache memory, where data are frequently accessed by the CPU. Unlike the smartphone Galaxy S10, which consists of three cache memories, the smartphone Galaxy A20E does not. Therefore, Galaxy A20E is less efficient, although the size of random access memory (RAM) of Galaxy A20E is large enough to host compressed models.

The integer quantized models had to be evaluated on ARM-based CPU devices (e.g., Android and iPhone devices) because the static execution plan was optimized to the integer arithmetic operator at conversion time. Therefore, when an x86-based CPU workstation attempts to process the quantized models, it conveys irregular computation patterns. For instance, the quantized Inception model at our server with Tesla V100 took more than an hour to process a single image.

We intentionally did not employ a mixture of augmentation because it did not make sense due to the characteristics of Gram-stained images. We refrained from applying scaling or any distortion techniques because magnification on a microscope is already fixed. Cropping is not allowed because it could easily trim out the microorganisms in the images. The color intensity of the images, however, might have been harmonized; nevertheless, we intentionally did not change color at default to increase variability and robustness because color is the most critical feature for Gram-stained analysis. Finally, employing a mixture of data augments in real time slowed the training time by a large margin.

Deep learning applications on the Internet of things (IoT) for healthcare create many opportunities because they can collect, harmonize and process data from multiple sources in real time. This will support caregivers to provide better treatments with lower cost at the right time. For instance, several successful applications were developed during the COVID-19 pandemic. Drew et al. [29] recruited about 2 million users and predicted geographical hotspots in advance of official public health reports. Alkhodari and Khandoker [30] developed COVID detection tools and demonstrated the potential of telehealthcare. However, there are still several challenges that need to be addressed. The disadvantages of IoT are security and privacy concerns due to the lack of holistic information security approaches for the IoT [31]. Cloud computing in healthcare has paved the way for rapid and low-cost healthcare services; however, the risk of healthcare data breaches has also been aroused. According to reference [32], 3912 data breach cases were confirmed in the healthcare domain from 2005 to 2019 in the United States. Hence, utilizing deep learning compression techniques and processing data in a local device could reduce the risk of data breach because data are not transmitted to the cloud server.

Compressed DL solutions were tested in general purpose devices only (smartphones) in this study. Although the smartphone is one type of device that can host an augmented reality application by overlaying information on the display incorporating a built-in camera, deploying the solution to a body-worn device such as smart glasses would be more intuitive because such a device is able to project information directly through an optical head-mounted display (OHMD). Kim and Choi [33] surveyed 57 academic papers on the applications of smart glasses and stated that smart glasses are most often used in the healthcare domain (*n* = 21, 37%). Evaluating the performance of caregivers with and without augmented reality would be an interesting prospective study. Google glass would be the choice of the device because the models developed in this study could be seamlessly deployed and evaluated on other android devices like Google glass. Beyond Gram-stained image classification, more complex experiments can be conducted. For instance, Zieliński et al. [34] classified 33 different genera and species of bacteria with 660 images, and genus-level image classification can be carried out with the same dataset used in this study. It would be interesting to see how the rapid DL methodology can improve the inference time compared to the published solutions.

## 5. Conclusions

Despite many publications proving the success of DL in medical applications, deploying a DL solution to resource constraint devices is a hard problem. This paper emphasized that DL models must be carefully designed with consideration of resource-limited devices. We investigated a rapid and compact DL model and evaluated the model performance on two mobile devices. The lessons learned and empirical guidelines drawn out of this study are as follows: we observed that the behavior and performance of models heavily rely on the tuning ratio but not on the batch size. For Gram-stained image classification, retraining more convolutional layers achieved higher accuracy. With respect to model compression, plain models were compressed successfully with minor or no accuracy loss. Pruning was the successful element for model size reduction, while inference time was mainly accelerated by quantization.

The philosophy of the collaboration of humans and computers shall be the right path for AI computers that amplify human competencies, not replace them. We anticipate that the rapid AR application of smart glasses or mobile devices can support caregivers for better and faster clinical decisions and can also be used for education purposes or assisting operations.

## Data Availability

The approval of the Data Protection office is currently in the works. As soon as we get approval, we will add data to the GitHub repository and update the readme file accordingly. In the meantime, we will provide the link to the DIBaS database, which is a publicly accessible Gram stain image dataset: https://github.com/gallardorafael/DIBaS-Dataset (accessed on 1 November 2022). Codes are available at: https://github.com/kimheekimi/rapid_gram_stain (accessed on 1 November 2022).

## Abbreviations

AI	Artificial intelligence
AR	Augmented reality
ASIC	Application-specific integrated circuit
BS	Batch size
CNN	Convolutional neural networks
DL	Deep learning
DNN	Deep neural networks
FDA	Food and Drug Administration
GPU	Graphics processing unit
IoT	Internet of things
mHealth	Mobile health
OHMD	Optical head-mounted display
RAM	Random access memory
TF	Transfer learning
TPU	Tensor processing unit
